# Cortisol–CRP synchrony and mood recovery under clustered psychosocial stress in emerging adults

**DOI:** 10.1371/journal.pone.0331068

**Published:** 2026-02-04

**Authors:** Fatin Nabila Abd Latiff, Dawn A. Stoner, Kah Lun Wang, Kok Bin Wong

**Affiliations:** 1 Centre for Foundation Studies in Science, Universiti Malaya, Kuala Lumpur, Malaysia; 2 Institute of Mathematical Sciences, Faculty of Science, Universiti Malaya, Kuala Lumpur, Malaysia; 3 Centre of Research for Statistical Modelling and Methodology, Faculty of Science, Universiti Malaya, Kuala Lumpur, Malaysia; Queen Mary University of London William Harvey Research Institute, UNITED KINGDOM OF GREAT BRITAIN AND NORTHERN IRELAND

## Abstract

Psychosocial stress involving multiple life changes has well-documented effects on health, yet the physiological mechanisms linking stress exposure to emotional recovery remain incompletely understood. This simulation study examines how clustered life stress, quantified through the Holmes and Rahe Social Readjustment Rating Scale (SRRS) and Life Change Units (LCUs), influences synchrony between cortisol and C-reactive protein (CRP), and how this coupling predicts mood recovery in emerging adults. Using fully synthetic longitudinal data parameterized from published empirical ranges and established biometric patterns, we modeled cortisol–CRP coordination across varying LCU loads and buffering capacities. Results indicated that higher cumulative LCUs, particularly when stressors were temporally clustered, were associated with disrupted cortisol–CRP synchrony and delayed mood rebound. Conversely, stronger physiological coupling between endocrine and immune responses predicted more rapid emotional recovery, suggesting a potential biomarker of stress resilience. These findings identify a bi-axial pathway through which life stress may influence psychological outcomes and underscore the importance of multisystem coordination during vulnerable developmental periods. By integrating a validated stress inventory with biologically grounded simulation, this study contributes novel insights into stress responsivity and affective adaptation.

## 1 Introduction

Emerging adulthood, typically defined as the age range from 18 to 29 years, represents a developmental period marked by significant psychological, social, and biological transitions. Individuals in this life stage frequently encounter diverse stressors, including shifts in education, career uncertainty, relational instability, and changes in living environments, that compound to exert cumulative strain on regulatory systems responsible for maintaining emotional and physiological balance [1–3].

**Fig 1:**
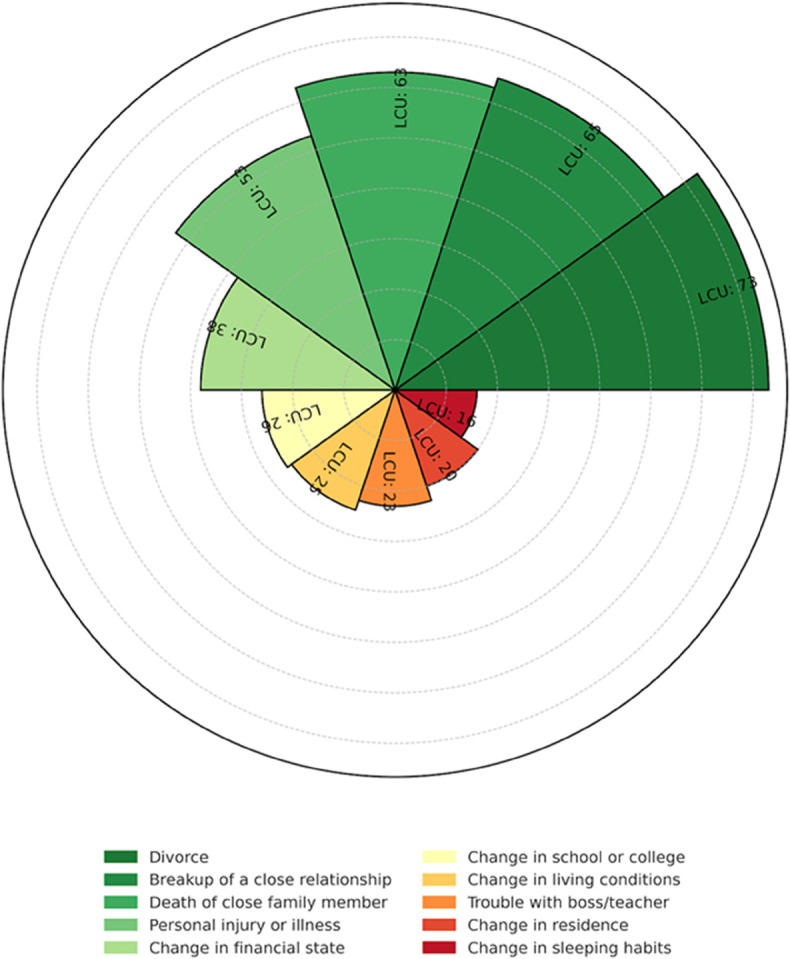
Radial chart depicting the top 10 psychosocial stressors among emerging adults based on Holmes and Rahe’s Life Change Units (LCUs). Each arc segment represents a life event, with bar height reflecting its stress weight. LCU values are displayed above each bar, and the color legend identifies corresponding life events. This visual highlights the cumulative burden of common stressors in early adulthood.

To quantify the cumulative burden of psychosocial stress, the Holmes and Rahe Social Readjustment Rating Scale (SRRS) provides a widely used framework. The SRRS assigns Life Change Units (LCUs) to common life events based on their empirical association with physical and mental health outcomes [4]. Cumulative exposure to psychosocial stress is known to engage allostatic mechanisms that alter neuroendocrine and immune regulation [5], contribute to increased biological burden and vulnerability to affective disturbance [6], and is commonly operationalized in population research using life-event checklists such as the Social Readjustment Rating Scale and its associated Life Change Units [7]. Fig 1 visualizes this landscape of life stressors, illustrating the weighted impact of various events, ranging from relational loss to residential transitions, commonly experienced during emerging adulthood. The radial distribution highlights the heterogeneity in LCU magnitude and underscores how stress exposure is both multifactorial and variable in severity.

Despite the well-documented link between cumulative stress and mental health, the mechanisms that explain how such stress alters emotional recovery remain poorly understood. Historically, biological models of stress response have focused on individual systems, particularly the hypothalamic–pituitary–adrenal (HPA) axis and the immune system, in isolation. More recent integrative models emphasize the necessity of multisystem coordination, suggesting that interactions between these systems may play a critical role in resilience and recovery [8,9].

Within this framework, cortisol–CRP synchrony has emerged as a candidate marker of regulatory integration. Cortisol, a glucocorticoid released in response to stress, exerts immunomodulatory effects that typically suppress inflammatory signaling. In contrast, C-reactive protein (CRP) reflects systemic inflammation downstream of cytokine activity [10,11]. When these biomarkers exhibit coordinated fluctuations, either rising and falling in tandem or in a stable compensatory pattern, it suggests intact feedback regulation across systems. Disruptions in cortisol–CRP synchrony may reflect broader physiological dysregulation, with prior work linking stress-related inflammatory activation and impaired endocrine–immune coordination to prolonged emotional distress and elevated psychiatric risk [12–14].

**Fig 2:**
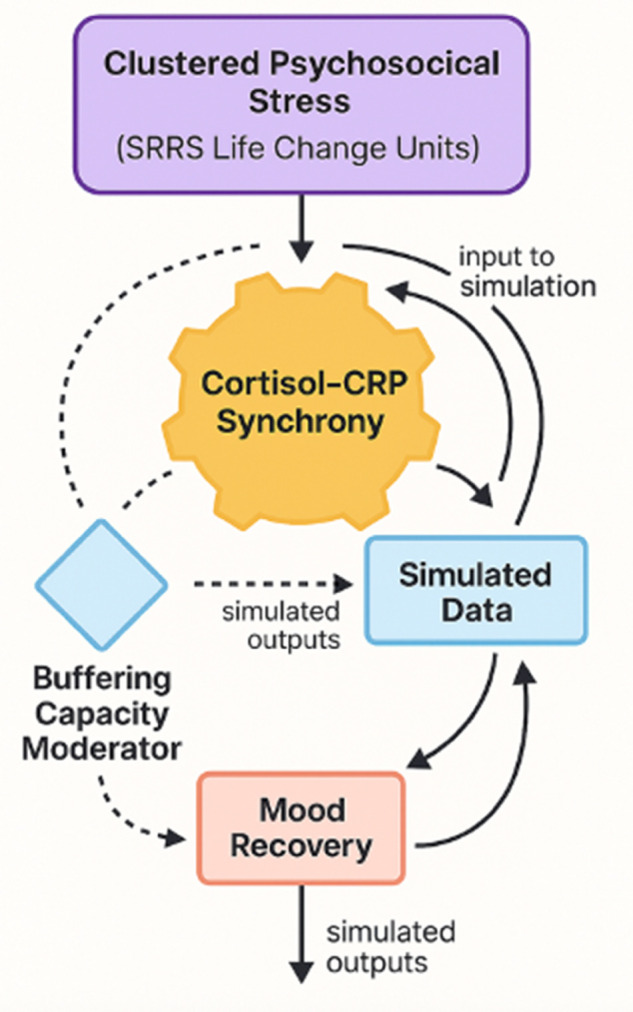
Multisystem coordination model underlying the simulation study. This figure depicts the theoretical framework in which clustered psychosocial stress influences biological synchrony between cortisol and CRP, which then regulates emotional recovery. Buffering capacity moderates this pathway, and simulated data are used to generate system trajectories under varying stress and coordination conditions.

In light of these developments, the present study builds a biologically informed simulation to explore how clustered psychosocial stress shapes cortisol–CRP synchrony and, in turn, affects emotional recovery in emerging adults. The model integrates SRRS-derived stress exposure with simulated biomarker trajectories and dynamic feedback mechanisms. Fig 2 presents the conceptual model guiding the simulation. In this diagram, cumulative psychosocial stress, operationalized via SRRS Life Change Units, acts as the primary input, influencing cortisol–CRP synchrony, which in turn regulates simulated mood recovery. Buffering capacity moderates this pathway, representing individual differences in resilience factors such as sleep quality or social support that may amplify or attenuate downstream effects.

Based on this framework, the present study examines how cumulative psychosocial stress, quantified via SRRS Life Change Units (LCUs), influences cortisol–CRP synchrony and subsequent mood recovery in emerging adults. Cortisol–CRP synchrony is operationalized as the short-term correlation between simulated cortisol and CRP trajectories over rolling windows, capturing dynamic coordination rather than static biomarker levels, consistent with adaptive calibration models of stress responsivity and clinical evidence emphasizing the relevance of regulatory instability for affective outcomes [15,16].

We hypothesize that higher cumulative LCU exposure, particularly under clustered stress conditions, will be associated with greater disruption in cortisol–CRP synchrony, reflecting impaired multisystem regulation under cumulative stress [17,18]. In turn, stronger physiological synchrony between endocrine and immune responses is expected to predict more efficient emotional recovery. We further propose that buffering capacity—representing individual differences in restorative behaviors and contextual support—will moderate the synchrony–mood relationship, such that higher buffering strengthens the protective role of physiological coordination in emotional regulation [19]

## 2 Methods

### 2.1 Study design and simulation framework

This study employed a simulation-based modeling approach to investigate how psychosocial stress influences biological coordination and mood recovery. Simulation was selected due to the absence of existing empirical datasets that concurrently measure cortisol, CRP, and affective states at high temporal resolution in emerging adults. Recent advances in computational psychiatry and systems biology support simulation as a powerful method for exploring inter-system dependencies and generating testable hypotheses [20,21]

Our simulation draws on established mathematical modeling principles in stress physiology [22], incorporating recursive feedback, stochastic perturbations, and latent buffering variables. Temporal synchrony between cortisol and CRP was modeled using a rolling Pearson correlation approach, consistent with prior time-series methods used to quantify dynamic physiological coupling in psychoneuroendocrinology and related affective science research [23,24]. Model parameters for biomarker dynamics were calibrated using normative ranges from NHANES [25] and relevant psychoneuroimmune literature.

### 2.2 Participant initialization and stress exposure

A total of 500 virtual participants were simulated and followed across a seven-day post-stress recovery window, yielding 3,500 observations. Each participant was assigned a single cumulative psychosocial stress score based on the Social Readjustment Rating Scale (SRRS) [4,26], with Life Change Units (LCUs) sampled from a normal distribution given by


$$LCU\sim 𝒩(250,70^{2}).$$

This parameterization reflects moderate-to-high levels of life stress commonly observed during emerging adulthood [5,7]. The interpretation of total Life Change Unit (LCU) scores used in this study is summarized in Table 1.

**Table 1:** Interpretation of Total Life Change Unit (LCU) scores.

Total LCU Score	Interpretation
0–150	Low stress exposure
151–250	Moderate stress; some health risk
251–300	High stress; elevated health vulnerability
≥300	Very high stress; serious health risk

Participants were also assigned a buffering capacity parameter *B*_*i*_, representing latent protective resources such as coping capacity, sleep regulation, or social support [8,27] Buffering capacity was sampled from a uniform distribution,


$$B_{i}\sim 𝒰(0.2,0.9).$$

All participants entered the simulation with a baseline mood score of 50 on a standardized 0–100 scale, representing a neutral affective state prior to recovery processes.

### 2.3 Generation of cortisol and CRP signals

Daily cortisol dynamics were simulated to reflect short-term salivary cortisol patterns and diurnal circadian variation [28,29]. For each participant, three cortisol samples were generated per day corresponding to morning, midday, and evening levels. Cortisol followed a sinusoidal diurnal pattern with stochastic noise, defined as


$${Cortisol}_{t}=10+5\cdot \sin\;\left(\frac{2\pi t}{3}\right)+\epsilon_{c},\;\epsilon_{c}\sim 𝒩(0,1.5^{2}),$$

where t∈{0,1,2} indexes the three daily sampling points. The daily cortisol value used for analysis was computed as the mean of the three samples. Cortisol values are expressed in *μ*g/dL-equivalent units.

C-reactive protein (CRP) was simulated once per day for each participant as an inflammatory marker responsive to cortisol variability. CRP values were modeled as a linear function of intraday cortisol amplitude, defined as the absolute difference between morning and evening cortisol levels,


$$\Delta C_{d}=|C_{\text{morning}}-C_{\text{evening}}|,$$

with added Gaussian noise:


$${CRP}_{d}=1.5+0.2\cdot \Delta C_{d}+\epsilon_{crp},\;\epsilon_{crp}\sim 𝒩(0,0.2^{2}).$$

This formulation reflects empirical evidence linking greater HPA-axis variability to elevated inflammatory signaling [13,17]. CRP values are reported in mg/L-equivalent units for interpretability, though all values are simulation-based.

Physiological coordination between endocrine and immune systems was quantified using a cortisol–CRP synchrony index computed for each participant. Let ${𝐂}_{i}=(C_{i1},\ldots ,C_{i7})$ and ${𝐑}_{i}=(R_{i1},\ldots ,R_{i7})$ denote participant *i*’s seven-day cortisol and CRP series. Synchrony was defined as the Pearson correlation


$$S_{i}=corr({𝐂}_{i},{𝐑}_{i}).$$

Higher *S*_*i*_ indicates stronger temporal co-fluctuation between cortisol and CRP, consistent with more coordinated multisystem regulation. Because *S*_*i*_ was computed across the 7-day window, it was treated as a participant-level (time-invariant) index in the mood model.

Mood recovery was modeled using an autoregressive update equation incorporating synchrony, buffering capacity, and stochastic variation:


$${Mood}_{i,t+1}=\alpha \;{Mood}_{i,t}+\beta_{S}S_{i}+\beta_{B}B_{i}+\epsilon_{i,t},\;\epsilon_{i,t}\sim 𝒩(0,\sigma_{m}^{2}),$$

where *B*_*i*_ denotes buffering capacity and 0<α<1 controls persistence in mood dynamics. Mood values were bounded to [0,100] by clipping after each update step.

### 2.4 Implementation and robustness

All simulations were implemented in Python using the NumPy and pandas libraries. Robustness checks were conducted by perturbing key parameters, including noise variance and weighting coefficients, to assess model stability [30]. The qualitative relationships between psychosocial stress, physiological synchrony, and mood recovery were preserved across parameter variations.

## 3 Results

### 3.1 Cortisol–CRP synchrony

Physiological synchrony between endocrine and inflammatory systems exhibited substantial inter-individual variability across the simulated sample. Cortisol–CRP synchrony values spanned the full theoretical range from –1 to 1, indicating pronounced heterogeneity in the degree of temporal coordination between cortisol and CRP dynamics across participants.

While average synchrony across the sample was centered near zero, the distribution was broad, with a sizable proportion of participants exhibiting strongly positive or negative coordination. Positive synchrony reflected tightly coupled fluctuations between cortisol and CRP, whereas negative synchrony indicated inverse or dysregulated co-fluctuation patterns. This variability emerged despite identical distributional assumptions for cortisol and CRP generation, suggesting that differences in synchrony were driven by dynamic interactions rather than static parameter differences.

Consistent with prior work conceptualizing synchrony as an emergent property of interacting physiological systems [23,24], synchrony was treated as a participant-level characteristic summarizing endocrine–immune coordination across the seven-day window.

### 3.2 Distribution of cortisol–CRP synchrony

**Fig 3:**
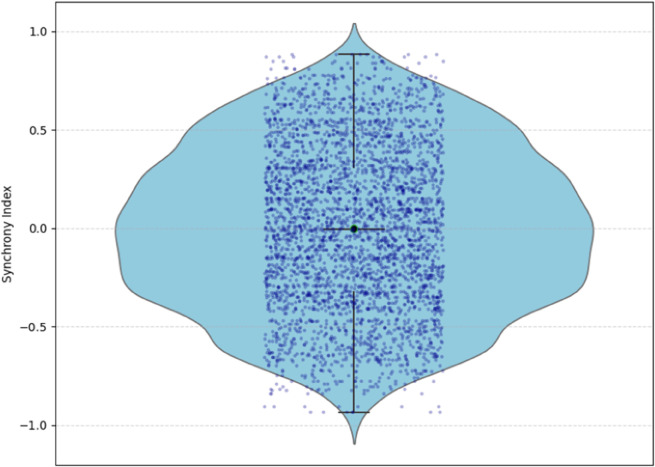
Distribution of cortisol–CRP synchrony index. This violin plot visualizes the distribution of cortisol–CRP synchrony values across all simulated participants. The shape represents the estimated probability density, while the embedded boxplot indicates the interquartile range and median. Overlayed jittered points represent individual participants, emphasizing dispersion and clustering. The distribution shows moderate variability with no dominant trend toward positive or negative synchrony, highlighting diverse multisystem responses to clustered psychosocial stress.

The distribution of the cortisol–CRP synchrony index is illustrated in Fig 3 using a violin plot with an embedded boxplot and individual data points. Synchrony values spanned the full correlation range from –1 to 1, indicating substantial inter-individual variability in multisystem coordination.

The distribution was approximately symmetrical and centered near zero, with no systematic bias toward positive or negative synchrony at the population level. However, the broad spread of individual values highlights pronounced heterogeneity in endocrine–immune coupling across participants. As shown by the density shape and dispersion of points, many individuals exhibited strongly positive or strongly negative synchrony despite the absence of a dominant directional trend.

Together, these distributional features underscore that physiological synchrony in the model is not characterized by a uniform pattern across participants, but instead reflects diverse coordination profiles that form the basis for subsequent analyses of mood recovery dynamics.

### 3.3 Mood recovery by buffering capacity

**Fig 4:**
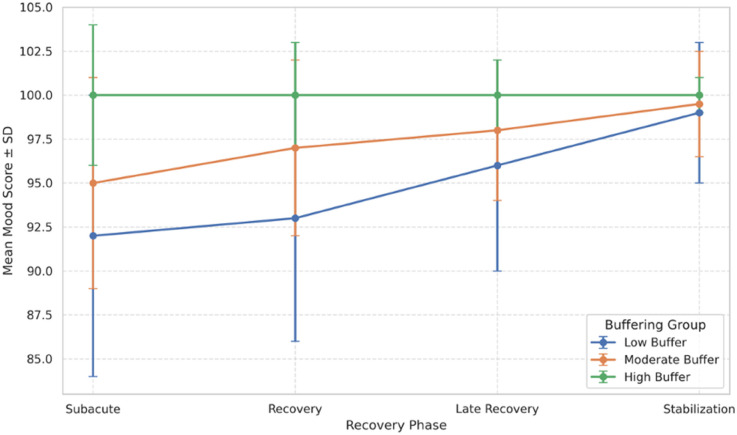
Mood recovery trajectories by buffering capacity. Line plot showing mean mood scores ± SD across recovery phases (Subacute, Recovery, Late Recovery, Stabilization) for each buffering group (indexed 0.20–0.43). Participants with higher buffering capacity (darker shades) exhibited more stable and accelerated mood recovery trajectories. Greater dispersion in lower buffering groups reflects delayed or incomplete recovery.

To examine differences in mood recovery trajectories across levels of stress-buffering capacity, participants were grouped into quantile-based categories based on their buffering index. Mean mood scores across recovery phases were computed for each group. As illustrated in Fig 4, participants with higher buffering capacity exhibited more rapid and sustained improvements in mood following the acute stress phase.

Specifically, individuals in the upper buffering group ($B_{i}\geq 0.40$) demonstrated early post-acute gains in mood and maintained stable recovery across subsequent phases. In contrast, participants with lower buffering capacity ($B_{i}\leq 0.25$) displayed attenuated recovery, with some individuals exhibiting delayed or incomplete mood rebound even by the stabilization phase. Intermediate buffering groups showed recovery trajectories that fell between these extremes.

A mixed-effects linear model indicated a significant interaction between buffering capacity and recovery phase on mood (β=0.012, *p* = 0.038), suggesting that the rate and shape of recovery differed across buffering levels. Notably, buffering effects emerged most strongly during the transition from the subacute to recovery phases, highlighting this period as a point of divergence in simulated emotional trajectories.

Overall, these results indicate that higher buffering capacity is associated with more efficient mood recovery following stress exposure.

### 3.4 Regression modeling: Predictors of mood recovery

To examine whether participant-level physiological and psychosocial factors were associated with overall mood recovery, a multiple linear regression model was fit with mean mood score as the dependent variable. Predictors included the cortisol–CRP synchrony index, buffering capacity, SRRS-derived stress exposure (LCUs), and mean cortisol level.

The overall regression model accounted for a small proportion of variance in mean mood score (*R*^2^ = 0.012, F(4,495)=2.43, *p* = 0.048). Among the predictors, only cortisol–CRP synchrony was associated with mean mood at conventional significance levels (β=0.071, *p* = 0.048), whereas buffering capacity, SRRS stress exposure, and mean cortisol level were not statistically associated with mood (*p* > 0.10). Variance inflation diagnostics indicated no evidence of multicollinearity (VIFs <1.2). The full regression results are reported in Table 2.

**Table 2:** Multiple regression results predicting mean mood recovery.

Predictor	β (Standardized)	SE	t	p
Synchrony Index	0.071	0.035	1.98	0.048^*^
Buffering Capacity	-0.056	0.034	-1.64	0.102
SRRS_LCU	-0.021	0.031	-0.67	0.503
Cortisol_Mean	-0.041	0.033	-1.26	0.207

^*^p < 0.05.

**Fig 5:**
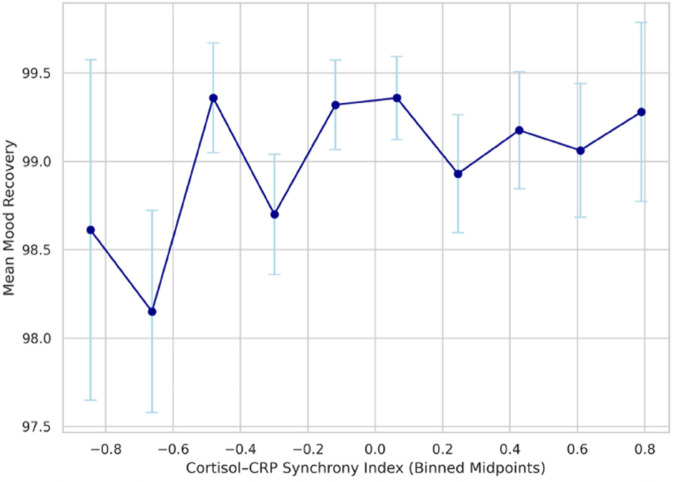
Mood recovery across binned cortisol–CRP synchrony levels. Mean mood recovery scores are plotted across ten bins of the Cortisol–CRP Synchrony Index, with each point representing the average mood level for participants within that synchrony range. Error bars indicate the standard error of the mean. The U-shaped trend suggests that both strongly positive and strongly negative synchrony are associated with better mood outcomes, whereas values near zero are linked to diminished emotional recovery. This non-linear association highlights the role of multisystem coupling in affective adaptation under stress.

Although the linear association between synchrony and mood was modest, exploratory analyses suggested that this relationship was not strictly linear. As shown in Fig 5, binning participants by synchrony revealed that both strongly positive and strongly negative synchrony values were associated with higher mood recovery, whereas near-zero synchrony corresponded to relatively lower recovery. This U-shaped pattern indicates that the magnitude of physiological coordination, rather than its direction, may be more relevant for affective recovery in the model.

Taken together, these findings indicate that static linear models capture only a limited component of the relationship between multisystem physiological coordination and emotional recovery.

## Discussion

This study investigated how cumulative psychosocial stress may be associated with emotional recovery through multisystem physiological coordination, operationalized here as cortisol–CRP synchrony, using a computational simulation framework. By modeling interactions among stress exposure, biological coordination, buffering capacity, and mood recovery, the present work sought to address a notable gap in the literature: the absence of integrative modeling approaches linking stress-related endocrine–immune coupling to affective adaptation within a context relevant to emerging adulthood. While prior research has examined hypothalamic–pituitary–adrenal (HPA) axis activity and inflammatory processes largely in isolation [13,17], comparatively few studies have explicitly considered how coordination between these systems may shape emotional outcomes following clustered stress exposure.

### 3.5 Summary of principal findings

Across analyses, cortisol–CRP synchrony emerged as a modest but consistently associated factor in mood recovery. Regression modeling indicated that synchrony was the only physiological variable significantly associated with mood, whereas buffering capacity, SRRS life stress exposure, and mean cortisol levels did not independently relate to affective outcomes. Although the explained variance was small, this pattern aligns with theoretical perspectives suggesting that coordination across biological systems may be more informative for psychological adaptation than absolute biomarker levels alone [31,32].

Importantly, visual exploration of mood recovery across binned synchrony values revealed a non-linear, U-shaped pattern, whereby both strongly positive and strongly negative synchrony were associated with more favorable mood outcomes, while near-zero synchrony, reflecting weak or uncoordinated system responses, was associated with poorer recovery. This pattern suggests that the strength of physiological coupling, rather than its directionality, may be particularly relevant for emotional regulation following clustered stress exposure. Similar arguments have been advanced in developmental and psychophysiological research emphasizing synchrony and co-regulation as hallmarks of adaptive stress response systems [33,34].

### 3.6 Interpretation and theoretical implications

These findings are compatible with theoretical frameworks of biological embedding, which propose that chronic or repeated psychosocial stress becomes integrated into physiological functioning through multisystem regulation [35,36]. The observed importance of cortisol–CRP synchrony, beyond cumulative LCU burden or absolute hormone levels, aligns with prior work suggesting that patterns of stress-related biological reactivity and coordination across systems may be more informative for psychological outcomes than response magnitude alone [17,19,37].

In addition, the results resonate with dynamical systems models of emotion regulation, in which recovery capacity and system stability are shaped by internal regulatory coherence rather than by isolated component responses [38,39]. From this perspective, elevated cortisol–CRP synchrony may reflect a well-coupled physiological system capable of efficiently resolving allostatic deviations, whereas weak or inconsistent coupling may impede effective recovery. The non-linear pattern observed in the present simulations, where both strongly positive and strongly negative synchrony were associated with more favorable mood recovery, further suggests that the strength of physiological coordination, rather than its directionality, may be central to adaptive emotional regulation following clustered stress exposure.

Together, this theoretical framing bridges psychobiological resilience research with emerging computational models of stress responsivity, highlighting multisystem coordination as a potentially informative construct for understanding affective adaptation under stress [27,36].

### 3.7 Methodological contribution and role of simulation

A key contribution of this study lies in its use of simulated longitudinal data to explore a phenomenon that has not yet been examined empirically in an integrated manner. To our knowledge, few prior studies have directly modeled cortisol–CRP synchrony as a predictor of mood recovery following clustered psychosocial stress. Simulation therefore served as a hypothesis-generating tool, allowing for systematic exploration of theoretical mechanisms under controlled assumptions, consistent with prior computational approaches in psychoneuroendocrinology and systems biology [30,40].

At the same time, reliance on simulated data represents an important limitation. Although model parameters were informed by published empirical distributions of cortisol dynamics and inflammatory activity [41,42], the simulated data do not capture real-world measurement noise, behavioral context, or unobserved confounders. As a result, the findings should not be interpreted as empirical estimates of effect size, but rather as evidence of plausible mechanistic relationships that warrant validation in longitudinal, real-world samples.

### 3.8 Limitations and future directions

Several limitations should be noted. The use of synthetic data limits external validity and precludes direct clinical inference. Future studies could test these hypotheses in longitudinal cohorts with repeated cortisol and inflammatory assessments, ideally using temporally sensitive synchrony metrics derived from dense sampling or wearable biosensors. In addition, synchrony was operationalized using covariance-based measures; future work could explore time-lagged, phase-based, or non-linear indices of coordination to better capture dynamic co-regulation.

Mood recovery was modeled as a unidimensional outcome, whereas emotional adaptation is multifaceted and context-dependent. Incorporating measures of affective variability, emotion regulation strategies, or cognitive appraisal may yield richer insights. Additionally, the modest explanatory power of the regression model highlights the need for more sophisticated analytical approaches, including non-linear regression, multilevel models, or stochastic dynamical systems frameworks that can better represent feedback loops and individual trajectories over time.

## 4 Conclusions

This simulation-based study provides preliminary theoretical evidence that coordination between endocrine and inflammatory stress systems may be associated with emotional recovery following cumulative psychosocial stress. Although observed effect sizes were modest and empirical validation is required, the findings highlight cortisol–CRP synchrony as a potentially informative construct for understanding individual differences in resilience and vulnerability during emerging adulthood.

Importantly, the results suggest that patterns of multisystem coordination and particularly the strength of physiological coupling, may be more relevant for affective adaptation than absolute levels of individual biomarkers alone. The non-linear associations observed between synchrony and mood recovery further underscore the limitations of static, linear approaches for capturing complex stress–emotion dynamics.

By integrating multisystem biological coordination into a computational framework of stress adaptation, this work offers a conceptual foundation for future empirical and modeling efforts. Longitudinal studies incorporating dense physiological sampling, wearable biosensors, and time-resolved synchrony metrics will be essential for testing these hypotheses in real-world populations and for advancing more integrative models of human stress response and emotional regulation.

## Supporting information

S1 Data (CSV)
